# Emerging Roles of Tripartite Motif-Containing Family Proteins (TRIMs) in Eliminating Misfolded Proteins

**DOI:** 10.3389/fcell.2020.00802

**Published:** 2020-08-25

**Authors:** Litian Zhang, Lukman O. Afolabi, Xiaochun Wan, Yang Li, Liang Chen

**Affiliations:** ^1^Shenzhen Laboratory of Tumor Cell Biology, Center for Antibody Drug Development, Institute of Biomedicine and Biotechnology, Shenzhen Institutes of Advanced Technology, Chinese Academy of Sciences, Shenzhen, China; ^2^Department of Hematology, Zhujiang Hospital, Southern Medical University, Guangzhou, China; ^3^University of Chinese Academy of Sciences, Beijing, China

**Keywords:** TRIMs, misfolded proteins, aggregates, degradation, UPS, autophagy, ERAD

## Abstract

Protein quality control (PQC) is pivotal for eukaryotic cells to eliminate misfolded proteins and maintain cellular homeostasis. A decreased or increased capacity of PQC is associated with various diseases, e.g., neurodegenerative diseases and cancers. Recently, increasing evidences have suggested that tripartite motif-containing family proteins (TRIMs) are the key players in PQC regulation. Most TRIMs are E3 ubiquitin ligases, such as TRIM11/19/25, which, through the ubiquitination modifications, can contribute to effectively remove the cellular misfolded proteins or protein aggregates via the UPS pathway. In this review, we summarized the participation of TRIM members in misfolded protein elimination through distinct pathways, including the ubiquitin–proteasome system (UPS), autophagy system, and ER-associated degradation (ERAD).

## Introduction

Proteins are the basic components of cells and are involved in a broad array of cellular processes. As part of the most abundant macromolecules, the cells inevitably need to put a huge strain on the protein production and maintenance of their natural conformations (Dobson, 2003). When cells are in certain physiological states or are exposed to various stress conditions, this leads to a condition in which the correct protein conformations are lost, leading to protein misfolding (Horwich and Weissman, 1997; Balch et al., 2008; Powers and Balch, 2008). Failure to timely remove the misfolded proteins can lead to the generation of proteotoxic stress (Bucciantini et al., 2002; Costanzo and Zurzolo, 2013; Soto and Pritzkow, 2018). Thus, maintaining cellular proteostasis is a requisite for cells to perform their basal function. To achieve this, the cell employs a fairly complex protein quality control (PQC) system that is critical to sequestrate, refold, and degrade any unexpected, accumulated misfolded proteins (Balchin et al., 2016; Sontag et al., 2017). The endoplasmic reticulum (ER) is an important cellular organelle that plays critical roles in the production, processing, and transport of proteins and lipids. It is also the organelle responsible for the maturation of roughly one-half proteins, in which aberrant proteins could be generated particularly under various physiological stress conditions (Wiseman et al., 2007; Wang and Kaufman, 2016). In ER, PQC is also known as ER quality control (ERQC) (Kim et al., 2015), for which nonnative conformational proteins can be refolded and modified following activation of the unfolded protein response (UPR) (Ron and Walter, 2007; Hetz et al., 2015) or eliminated *via* ER-associated degradation (ERAD) (Hiller et al., 1996). Studies have shown the selective degradation of harmful or exhausted organelles via a specific type of autophagic turnover such as ER-phagy. When the ER becomes overwhelmed and stressed, its fragmented components along with the aberrant protein are delivered to the lysosome where they are degraded via ER-phagy (Grumati et al., 2018).

The cell’s PQC system consists of two separate but collaborated parts: (I) molecular chaperone system, which is constituted by various types of heat shock proteins (HSPs) that function to release and unfold individual misfolded proteins from aggregates (Hartl and Hayer-Hartl, 2002; Sharma et al., 2008; Kim et al., 2013); (II) the degradation system, which relies on the ubiquitin–proteasome system (UPS) and autophagy pathways (Goldberg, 2003; Finley, 2009; Wani et al., 2015). In particular, molecular chaperones – a class of protein family that are evolutionarily conserved and are widely distributed in various organisms – are essential for cell survival, including HSP60, HSP70, HSP100, small HSP, and calnexin (Richter et al., 2010). When a protein is misfolded, molecular chaperones assist in the correct folding of the misfolded protein by reversibly binding to stabilize the unstable intermediates, followed by its release and refolding to its native conformation.

Meanwhile, erroneous protein aggregates that cannot be refolded can be disaggregated by chaperones as well (Saibil, 2013). Molecular chaperone system can also be overstressed, and in such condition, it directs the inundated misfolded proteins or protein aggregates to cellular clearance pathways via the ubiquitin–proteasome pathway or sequestration in autophagosomes (Kaganovich et al., 2008). The UPS and autophagy systems represents two distinct, selective, and well-regulated cellular degradative pathways, with their respective subcellular localization, mechanisms, machinery, and degradative substrates (Mishra et al., 2018). Emerging evidences have shown that these two systems have cross-talk through ubiquitination (Varshavsky, 2017; Goodier et al., 2020), implying that a complementary and synergistic function of the UPS and autophagy systems may exist (Korolchuk et al., 2009; Kwon and Ciechanover, 2017). In addition, these pathways – alone or in cooperation with each other – orchestrate the entire intracellular protein degradation (Wong and Cuervo, 2010; Chhangani et al., 2014).

Ubiquitination is accomplished by three enzymatic steps catalyzed by (1) ubiquitin-activating enzymes (E1s), (2) ubiquitin-conjugating enzymes (E2s), and (3) ubiquitin ligases (E3s). However, the specificity and efficiency of this system (protein ubiquitylation) are largely determined by the E3 ubiquitin ligases that recognize specific substrates (Zheng and Shabek, 2017). Misfolded proteins can be degraded upon the covalent attachment of ubiquitin. Ubiquitination can be either monoubiquitination (addition of a single ubiquitin molecule) or polyubiquitination (addition of a chain of ubiquitin molecules), and the fate of ubiquitinated substrates is determined by the position of the lysine by which polyubiquitination is mediated through K11, K48, K63, etc. (Hjerpe and Rodriguez, 2008; Xu et al., 2009; Sadowski et al., 2012).

For example, the K48-polyubiquitinated substrates are prone to be eliminated by UPS (Grice and Nathan, 2016), while the K63-polyubiquitinated or monoubiquitinated substrates undergo elimination by autophagy (Sun et al., 2018). Hence, the structural complexity of distinct polyubiquitin chains is sufficient to maintain the selectivity and specificity of the UPS and autophagy for each substrate (Alfano et al., 2016). This also suggests that substrates can be recognized through polyubiquitin chains of different topologies, providing degradation signals for distinct protein degradation pathways (Ohtake et al., 2016). Therefore, researches focused on ubiquitin-related enzymes have gained much attention.

**FIGURE 1:**
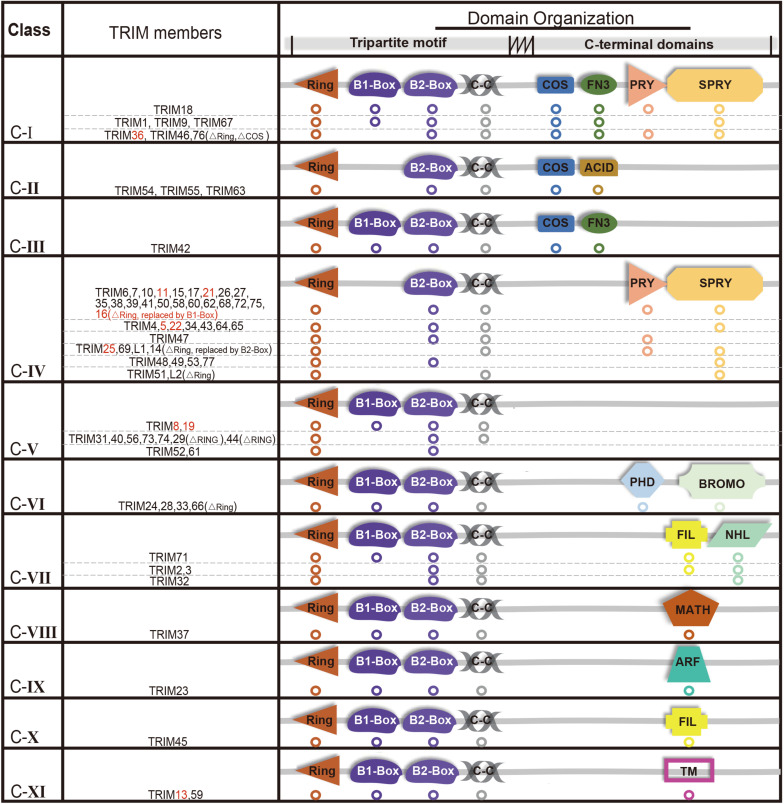
Structure and classification of TRIMs (those mediating degradation of misfolded proteins are marked in red, which are also elaborated in this review). Based on the secondary domain organization, TRIMs are categorized into 11 distinct subgroups (C-I to C-XI). Among 10 TRIMs that are summarized in this review, over one half belongs to C-IV, and sporadic ones to C-I, C-V, or C-XI, respectively. The majority of TRIMS has a highly conserved tripartite motif (RBCC) at N-terminus, while a small number are still missing RING domain. The abbreviations of C-terminal domains are listed as follows: C-terminal subgroup One Signature domain (COS), fibronectin type III repeat domain (FN3), PRY domain, SPRY domain, acid-rich region (ACID), filamin-type IG domain (FIL), NHL domain, PHD domain, bromodomain (BROMO), Meprin and TRAF-homology domain (MATH), ADP-ribosylation factor family domain (ARF), and transmembrane region (TM). Besides, “ΔRING” means the RING domain is absent.

The E3 ligases have a large number and has been extensively studied compared with a small number of E1s and E2s (Deshaies and Joazeiro, 2009; Berndsen and Wolberger, 2014; Buetow and Huang, 2016). TRIMs belong to E3s and over 70 members of the TRIMs have been identified in humans and mice (Hatakeyama, 2011). Most of the TRIMs consist of a highly conserved tripartite motif at N-terminus: a RING domain, one or two B-box domain, and a coiled-coil domain (Meroni and Diez-Roux, 2005; James et al., 2007; Li et al., 2014). The RING domain exhibits ubiquitin E3 ligase activity, yet there is still a limited number of TRIMs that are RING-deficient proteins. TRIM proteins can generally form homopolymers and heteropolymers with each other through their coiled-coil domain. The B-box domains are characterized as a universal domain in TRIMs, while their C-terminal domains provide TRIMs diversities. Based on their domain organization, TRIMs are categorized into 11 distinct subgroups (C-I to C-XI) (Figure 1).

Tripartite motif-containing family proteins play significant roles in various physiological or pathological conditions by acting as ubiquitin E3 ligases, such as cell proliferation and development, DNA damage and repair, neurodegenerative disease, innate immune response, and carcinogenesis (Nisole et al., 2005; Ozato et al., 2008; Hatakeyama, 2017). The mechanisms of TRIMs in misfolded protein clearance remain mysterious, even though it is likely a key function in many of their biological effects. Our group has demonstrated that several TRIM members, including TRIM11/5/25, are crucial for eliminating misfolded proteins (Chen et al., 2017, 2018; Liu et al., 2020). TRIM11 mediates the degradation of misfolded proteins and protein aggregates in the nucleus and cytoplasm through regulating UPS. TRIM5 may mediate protein aggregate degradation through autophagy, and TRIM25 mediates the degradation of misfolded proteins in the ER through ERAD. Other investigators discovered that TRIM13, an ER transmembrane (TM)-anchored E3 ligase, also mediates ERAD particularly for some membrane and secretory proteins from the ER (Tomar et al., 2012). TRIM13 can also orchestrate the initiation of ER-phagy via the N-degron pathway (Ji et al., 2019).

**FIGURE 2:**
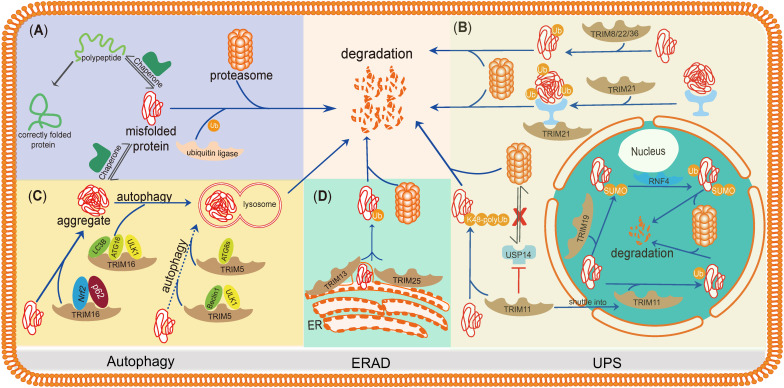
TRIMs mediate degradation of misfolded proteins or protein aggregates through the UPS, autophagy, and ERAD. TRIMs are widely involved in misfolded proteins clearance through distinct mechanisms, and some of them even play dual roles and function in different organelles. **(A)** The basic mechanism of degradation of misfolded proteins in PQC. **(B)** TRIM19 (PML), as well as TRIM11, participates in degradation of misfolded proteins in the nucleus via UPS, while the latter also works in cytosol by enhancing the activity of proteasome. TRIM21, as a cytoplasmic Fc receptor, is of high affinity to the complexes composed by aggregates and specific antibody and then activates UPS. Besides, TRIM8/22/36 are also supposed to promote misfolded proteins’ degradation through UPS. **(C)** TRIM16 promotes misfolded proteins into aggregates by interacting with Nrf2 and p62 and then enhances the biogenesis of autophagosome and autophagy-mediated aggregate removal. TRIM5 acts as an autophagic scaffold protein for Beclin1 and ULK1, which interacts with Atg8s for selective autophagy. **(D)** Upon ER stress, TRIM25 triggers ERAD to degrade misfolded proteins through ROS-associated pathways potentially. TRIM13, a membrane-bound protein of ER, also contributes to ERAD with its transmembrane domain.

Here, we summarize the studies of TRIMs in misfolded protein clearance, regarding how TRIMs regulate their downstream pathways and whether they function synergistically or compensate each other. The purpose of this mini-review is to highlight the roles of TRIMs in misfolded protein degradation and describe the internal connections of TRIMs during these processes (Figure 2).

## TRIMs in Eliminating Misfolded Proteins via UPS

### TRIM19

TRIM19, a typical RBCC ligase without any C-terminal domains, is well known as the promyelocytic leukemia protein (PML), which recruits diverse proteins to assemble PML nuclear bodies (NBs) (Nisole et al., 2013). It has been confirmed that PML NBs are involved in different cellular processes, e.g., antiviral response, DNA damage repair, and PQC (Bernardi and Pandolfi, 2007), implying a potential role of TRIM19 in removing misfolded proteins.

A polyQ stretch expansion in the nucleus of spinal or cerebellar neurons is pathologically associated with one of the neurodegenerative diseases spinocerebellar ataxias (SCAs) (Janer et al., 2006). SCA1 gene Ataxin-1 encodes pathogenic protein Atxn1-82Q, but not the normal protein Atxn1-30Q, forming protein aggregates in the nucleus, which could be co-localized and decreased by TRIM19 (Guo et al., 2014). Notably, more distinct sites on TRIM19 were identified to directly recognize multiple misfolded proteins [i.e., the CC region of the TRIM/RBCC motif (SRS1) and the 63 amino acid residues at its C terminus (SRS2)] in the nucleus. Mechanistically, TRIM19 first acts as a small ubiquitin-like modifier (SUMO) E3 ligase by conjugating SUMO to misfolded proteins, which are recognized and ubiquitinated by a SUMO-targeted ubiquitin ligase called RING finger protein 4 (RNF4). Through the sequential SUMOylation and ubiquitination, misfolded proteins in cancer cells are efficiently degraded via the proteasome (Guo et al., 2014); furthermore, the capacity of TRIM19 to mediate the degradation of Atxn1-82Q protein aggregates in differentiated acute promyelocytic leukemia (APL) cells was found to be markedly reduced (Chen et al., 2017). Thus, the function of TRIM19 provides a new insight, suggesting that maintenance of malignant phenotypes may rely on effective clearance of misfolded proteins.

### TRIM11

TRIM11 is structurally characterized by a typical TRIM/RBCC (RING, B-box, and coiled-coil) motif at its N-terminus and a PRY/SPRY domain at its C-terminus. Extensive studies have demonstrated the critical roles of TRIM11 in the processes of neurogenesis as well as oncogenesis (Niikura et al., 2003; Tuoc and Stoykova, 2008; Wang et al., 2016).

The expression of all TRIM genes in the human mammary epithelial cell (HMEC) transformation model was analyzed, and the results revealed that TRIM11 is amplified in a series of cancer cell lines and tissues, e.g., breast carcinoma, gliomas, and lung cancer. TRIM11 promotes the degradation of misfolded and aggregated proteins in tumor cells’ nucleus or cytosol; for instance, forced expression of TRIM11 was shown to reduce the inclusions formed by Atxn1-82Q and Httax1p-97QP (Chen et al., 2017).

Notably, the RING domain of TRIM11 is required to perform its functions. On one hand, this domain catalyzes the conjugation of K48-polyubiquitin to various types of substrates, thus, ensuring adequate concentration of substrate for UPS (Chen et al., 2017, 2018). On the other hand, it directly enhances the proteasome activity by interacting with the ubiquitin-like (UBL) domain of ubiquitin-specific protease 14 (also called USP14, which removes ubiquitin chains from substrates) which deterred the interaction between USP14 and the 19S subunit of the proteasome (Chen et al., 2018). Collectively, these studies revealed that TRIM11 effectively clears misfolded proteins through promoting the activity of proteasome.

### TRIM21

TRIM21/Ro52 is another member of the TRIM protein family that possesses a PRY/SPRY domain at its C-terminus, along with a typical RBCC motif at the N-terminus modulating its ubiquitin E3 ligase activity (Takahata et al., 2008). Reports have shown that TRIM21 has a high affinity to immunoglobulins (Keeble et al., 2008); thus, it acts mainly as an Fc receptor in the cytoplasm, to recognize the antigen–antibody complex (Kimura et al., 2015; Bottermann et al., 2018).

During immune defensive response against viral or bacterial infection, TRIM21 triggers pathogen neutralization by mediating UPS and valosin-containing protein (VCP, a molecular unfoldase) to eliminate such pathogens (McEwan et al., 2012). Similar to pathogen infection, misfolded tau proteins can be assembled, transferred, and spread in neurons leading to neuron cell apoptosis and induction of neurodegenerative disease. The accumulation of tau proteins has been associated with both acute neurological dysfunctions such as traumatic brain injury (TBI) and chronic neurodegenerative disorders like Alzheimer’s disease (Bloom, 2014; Edwards et al., 2020).

In addition, robust cis p-tau proteins that are found in TBI models were targeted and ablated by monoclonal antibodies (mAbs) (Kondo et al., 2015). These mAbs gained entry into the neurons via TRIM21 and then neutralized the tau aggregates, which was accompanied by TRIM21 recruitment (McEwan et al., 2017). Therefore, it inspires us to consider whether there are more similarities between invasion of pathogen and aggregation of misfolded proteins, and it raises our interest in investigating the critical roles of TRIM21 in several neuron system diseases via UPS.

### TRIM8, TRIM22, and TRIM36

TRIM8, TRIM22, and TRIM36 belong to a different subclass of TRIM family. They can be significantly upregulated during cell transformation and effectively promote the elimination of misfolded proteins in transformed cells (Chen et al., 2017). This class of TRIMs was observed to be upregulated along with the proteasome in tumor cells during cell transformation (Chen et al., 2017). Hence, we propose that TRIM8/22/36 enhance misfolded protein degradation probably through the UPS pathway; however, the mechanism through which they perform this function needs further investigation.

## TRIMs in Eliminating Misfolded Proteins/Aggregates via Autophagy

### TRIM16

TRIM16 (also termed as estrogen-responsive B box protein-EBBP) has a missing RING domain at the N-terminus but harbors two B-box domains that possess RING-like folds, which still has the E3 ligase activity, a coiled-coil region, and a C-terminal PRY-SPRY domain.

TRIM16 was reported to bolster the turnover of stress-induced misfolded proteins by positively regulating the nuclear factor erythroid 2-related factor 2 (Nrf2) and autophagy signaling pathways (Jena et al., 2018b, 2019). TRIM16 binds to Nrf2, inducing K63-ubiquitination of Nrf2 and enhancing p62-Kelch-like ECH-associated protein 1 (KEAP1) interaction, which displaces Nrf2 from KEAP1 and prevents Nrf2 from proteasomal degradation (Jena et al., 2018a). As a positive feedback loop, the activated Nrf2 then converts misfolded proteins into aggregates by induction of p62, TRIM16, and other ubiquitin system genes. Recent studies demonstrated that TRIM16 is capable of assembling the autophagy machinery that contains TRIM16 (which acts as a scaffold protein), unc-51 Like Autophagy Activating Kinase 1 (ULK1), ATG16, and LC3B, which governed autophagosome biogenesis, and thus accelerate protein aggregates sequestration and clearance (Jena et al., 2018a; New and Thomas, 2019). Taken together, TRIM16 acts as a central player in eliminating misfolded proteins/protein aggregates during exposure to oxidative/proteotoxic stress by modulating the autophagy pathway.

### TRIM5

TRIM5 has a similar structure to TRIM11 except for the absence of a PRY domain at its C-terminus. As an innate immune activator and a ubiquitin E3 ligase, TRIM5 is highly relevant in retroviral restriction such as anti-HIV infection (Pertel et al., 2011; Fletcher et al., 2018).

TRIM5 can be significantly upregulated during cell transformation to eliminate misfolded proteins/aggregates effectively (Chen et al., 2017). Mandell et al. (2014) screened all TRIMs involved in autophagy and identified that TRIM5 acts as a scaffold protein that interacts with the key members of autophagy like protein ULK1 and Beclin1. TRIM5 also served as a selective autophagic receptor mediating substrates’ autophagic degradation (Mandell et al., 2014). Thus, TRIM5 promotes the removal of misfolded proteins/aggregates most likely via the autophagy pathway.

## TRIMs in Eliminating Misfolded Proteins via ERAD

### TRIM25

TRIM25, known as the estrogen-responsive finger protein (EFP), possesses a typical N-terminal TRIM/RBCC motif and a C-terminal PRY/SPRY domain that has been extensively studied in innate immunity (Zou and Zhang, 2006; Versteeg et al., 2013). Previous studies showed that TRIM25 mainly acted as an E3 ligase and mediated ubiquitination of various key molecular biomarkers such as retinoic acid-inducible gene 1 (RIG-I) for antiviral response and peroxisome proliferator-activated receptor-gamma (PPARγ) for metabolism (Gack et al., 2007; Lee et al., 2018).

Since the ER is fundamental for protein and lipid biosynthesis as well as protein posttranslational processing and transportation, various physiological or pathological processes can lead to an accumulation of misfolded proteins in the ER resulting in a condition called ER stress (Wu and Kaufman, 2006). To cope with ER stress, cells rely on ERQC systems such as the UPR and ER-associated protein degradation (ERAD) (Guerriero and Brodsky, 2012; Hetz and Saxena, 2017). Recently, our group identified that TRIM25 is significantly induced upon ER stress, which promotes ERAD and finally restores ER homeostasis (Liu et al., 2020). Moreover, we found that TRIM25 expression is associated with hepatocellular carcinoma (HCC) progression and high TRIM25 expression correlates with poor patient survival in HCC (Liu et al., 2020). This reveals that TRIM25 modulates the ER homeostasis and could serve as a potential target for HCC therapy. In summary, TRIM25 is a novel ERQC player, mediating misfolded protein clearance via the ERAD.

### TRIM13

TRIM13 is another member of the TRIM protein family that is encoded by the Ret Finger Protein 2 (RFP2) gene that consists of RING, B-box, and coiled-coil domains. Besides, TRIM13 also contains a TM domain, which is required for its ER localization (Lerner et al., 2007). Interestingly, this TM domain is only found in TRIM13 and TRIM59 among all the TRIM members.

Reports have shown that TRIM13 can recognize ERAD substrate CD3δ and degraded it via UPS, which relies on its RING domain (Lerner et al., 2007). In a condition of ER stress, TRIM13 also contributes to autophagosome biogenesis through its strong interaction with p62 and double FYVE-containing protein 1 (DFCP1). The deletion construct of the coiled-coil domain of TRIM13 represses autophagy regulation (Tomar et al., 2012). Additionally, TRIM13 has been found to regulate the activation of caspase-8 to induce autophagy, leading to tumor cell death in tumors (Tomar et al., 2013). These studies enrich our understanding of the critical roles played by TRIM13 in ERAD and suggest that TRIM13 may be a tumor suppressor.

## Discussion and Future Prospects

In this review, we summarized the defined roles of TRIM proteins in misfolded protein clearance. These TRIM members can be localized at the nucleus, cytoplasm, or ER and facilitate the degradation of misfolded and aggregated proteins through distinct pathways, including the UPS, autophagy, and ERAD. Recent studies revealed that TRIM5 and TRIM16, serve as autophagy receptors for cargo recruitment providing the possibility that TRIM members can collaborate to regulate autophagy-mediated misfolded proteins’ clearance (Kimura et al., 2017; New and Thomas, 2019). In previous studies, TRIM5 has been shown to shuttle into the nucleus, where it forms heterodimer with TRIM19 (PML) or TRIM16 resulting in PML NBs (Stremlau et al., 2004; Diaz-Griffero et al., 2011; Bell et al., 2012); thus, indicating a synergistic effect of autophagy and UPS on misfolded protein removal. In addition, the complex of MAGE-A3/6-TRIM28 could target 5′ AMP-activated protein kinase (AMPK) for ubiquitination and proteasome-mediated degradation resulting in significant autophagy inhibition (Pineda et al., 2015). Together, these reports suggest a positive feedback loop and the existence of a cross-talk between the UPS and autophagy pathways. Thus, it is imperative to investigate the cooperation among the TRIM protein members in the degradation of misfolded protein via the UPS, autophagy, or their combination.

Of note, TRIM proteins not only act as E3 ligases but also may have extensive, yet unidentified roles. For example, TRIM19 (PML) possesses SUMOylation ligase activity in the nucleus (Guo et al., 2014), and other TRIMs with a SUMOylation ligase activity include TRIM5, TRIM27, and TRIM36 (Chu and Yang, 2011). Misfolded proteins may undergo sequential SUMOylation and ubiquitination mediated by one or several TRIM members. Moreover, the association between SUMOylation and ubiquitination and their roles in misfolded protein degradation provides a better understanding of those TRIMs containing TRIM19-like RBCC structure and is worth further exploration (Wang et al., 2018).

We have proven that the effective removal of misfolded proteins is required for oncogenesis and that TRIM members (like TRIM11) are upregulated and play critical roles during oncogenic transformation (Chen et al., 2017); this suggests that TRIM proteins can serve as novel therapeutic targets for clinical treatment of cancer. An approach has recently been developed to acutely and rapidly degrade endogenous proteins, which is based on TRIM21 and UPS pathway (Clift et al., 2017, 2018). This method, which can also precisely target the aberrant proteins in the cell, may contribute to developing other novel therapeutics for diseases associated with misfolded proteins such as cancers and neurodegenerative diseases.

To conclude, TRIM proteins are involved in misfolded protein clearance through distinct mechanisms, and the identification of other TRIM members capable of efficient misfolded proteins removal is imperative. More importantly, there exists a strong connection between TRIM proteins and proteostasis – a condition that can affect tumorigenesis and neurodegeneration. It is of key importance to further elucidate the relationships and dynamics between TRIMs and proteostasis as this is crucial for the development of novel therapeutic intervention by targeting relevant TRIMs in cancer, neurodegenerative diseases, and other associated pathologies.

## Author Contributions

LZ, LA, XW, YL, and LC contributed to the acquisition of data, interpretation of data, and drafting the article. LZ and LC contributed to analyze the data and wrote the manuscript. All authors contributed to the manuscript revision and read and approved the submitted version.

## Conflict of Interest

The authors declare that the research was conducted in the absence of any commercial or financial relationships that could be construed as a potential conflict of interest.

## References

[B1] Alfano C. Faggiano S. Pastore A. (2016). The Ball and Chain of Polyubiquitin Structures. *Trends Biochem. Sci.* 41 371–385. 10.1016/j.tibs.2016.01.006 26899455

[B2] Balch W. E. Morimoto R. I. Dillin A. Kelly J. W. (2008). Adapting proteostasis for disease intervention. *Science* 319 916–919. 10.1126/science.1141448 18276881

[B3] Balchin D. Hayer-Hartl M. Hartl F. U. (2016). In vivo aspects of protein folding and quality control. *Science* 353:aac4354. 10.1126/science.aac4354 27365453

[B4] Bell J. L. Malyukova A. Holien J. K. Koach J. Parker M. W. Kavallaris M. (2012). TRIM16 acts as an E3 ubiquitin ligase and can heterodimerize with other TRIM family members. *PLoS One* 7:e37470. 10.1371/journal.pone.0037470 22629402 PMC3357404

[B5] Bernardi R. Pandolfi P. P. (2007). Structure, dynamics and functions of promyelocytic leukaemia nuclear bodies. *Nat. Rev. Mol. Cell Biol.* 8 1006–1016. 10.1038/nrm2277 17928811

[B6] Berndsen C. E. Wolberger C. (2014). New insights into ubiquitin E3 ligase mechanism. *Nat. Struct. Mol. Biol.* 21 301–307. 10.1038/nsmb.2780 24699078

[B7] Bloom G. S. (2014). Amyloid-beta and tau: the trigger and bullet in Alzheimer disease pathogenesis. *JAMA Neurol.* 71 505–508. 10.1001/jamaneurol.2013.5847 24493463 PMC12908160

[B8] Bottermann M. Foss S. van Tienen L. M. Vaysburd M. Cruickshank J. O’Connell K. (2018). TRIM21 mediates antibody inhibition of adenovirus-based gene delivery and vaccination. *Proc. Natl. Acad. Sci. U.S.A.* 115 10440–10445. 10.1073/pnas.1806314115 30209217 PMC6187179

[B9] Bucciantini M. Giannoni E. Chiti F. Baroni F. Formigli L. Zurdo J. (2002). Inherent toxicity of aggregates implies a common mechanism for protein misfolding diseases. *Nature* 416 507–511. 10.1038/416507a 11932737

[B10] Buetow L. Huang D. T. (2016). Structural insights into the catalysis and regulation of E3 ubiquitin ligases. *Nat. Rev. Mol. Cell Biol.* 17 626–642. 10.1038/nrm.2016.91 27485899 PMC6211636

[B11] Chen L. Brewer M. D. Guo L. Wang R. Jiang P. Yang X. (2017). Enhanced Degradation of misfolded proteins promotes tumorigenesis. *Cell Rep.* 18 3143–3154. 10.1016/j.celrep.2017.03.010 28355566 PMC5603913

[B12] Chen L. Zhu G. Johns E. M. Yang X. (2018). TRIM11 activates the proteasome and promotes overall protein degradation by regulating USP14. *Nat. Commun.* 9:1223. 10.1038/s41467-018-03499-z 29581427 PMC5964324

[B13] Chhangani D. Chinchwadkar S. Mishra A. (2014). Autophagy coupling interplay: can improve cellular repair and aging? *Mol. Neurobiol.* 49 1270–1281. 10.1007/s12035-013-8599-z 24385255

[B14] Chu Y. Yang X. (2011). SUMO E3 ligase activity of TRIM proteins. *Oncogene* 30 1108–1116. 10.1038/onc.2010.462 20972456 PMC3103664

[B15] Clift D. McEwan W. A. Labzin L. I. Konieczny V. Mogessie B. James L. C. (2017). A method for the acute and rapid degradation of endogenous proteins. *Cell* 171 1692–1706.e1618. 10.1016/j.cell.2017.10.033 29153837 PMC5733393

[B16] Clift D. So C. McEwan W. A. James L. C. Schuh M. (2018). Acute and rapid degradation of endogenous proteins by Trim-Away. *Nat. Protoc.* 13 2149–2175. 10.1038/s41596-018-0028-23 30250286

[B17] Costanzo M. Zurzolo C. (2013). The cell biology of prion-like spread of protein aggregates: mechanisms and implication in neurodegeneration. *Biochem. J.* 452 1–17. 10.1042/BJ20121898 23614720

[B18] Deshaies R. J. Joazeiro C. A. (2009). RING domain E3 ubiquitin ligases. *Annu. Rev. Biochem.* 78 399–434. 10.1146/annurev.biochem.78.101807.093809 19489725

[B19] Diaz-Griffero F. Gallo D. E. Hope T. J. Sodroski J. (2011). Trafficking of some old world primate TRIM5alpha proteins through the nucleus. *Retrovirology* 8| :38. 10.1186/1742-4690-8-38 21575157 PMC3120760

[B20] Dobson C. M. (2003). Protein folding and misfolding. *Nature* 426 884–890. 10.1038/nature02261 14685248

[B21] Edwards G. III Zhao J. Dash P. K. Soto C. Moreno-Gonzalez I. (2020). Traumatic brain injury induces tau aggregation and spreading. *J. Neurotrauma* 37 80–92. 10.1089/neu.2018.6348 31317824 PMC6921297

[B22] Finley D. (2009). Recognition and processing of ubiquitin-protein conjugates by the proteasome. *Annu. Rev. Biochem.* 78 477–513. 10.1146/annurev.biochem.78.081507.101607 19489727 PMC3431160

[B23] Fletcher A. J. Vaysburd M. Maslen S. Zeng J. Skehel J. M. Towers G. J. (2018). Trivalent RING Assembly on Retroviral Capsids Activates TRIM5 Ubiquitination and Innate Immune Signaling. *Cell Host Microbe* 24 761–775.e766. 10.1016/j.chom.2018.10.007 30503508 PMC6299210

[B24] Gack M. U. Shin Y. C. Joo C. H. Urano T. Liang C. Sun L. (2007). TRIM25 RING-finger E3 ubiquitin ligase is essential for RIG-I-mediated antiviral activity. *Nature* 446 916–920. 10.1038/nature05732 17392790

[B25] Goldberg A. L. (2003). Protein degradation and protection against misfolded or damaged proteins. *Nature* 426 895–899. 10.1038/nature02263 14685250

[B26] Goodier J. L. Soares A. O. Pereira G. C. DeVine L. R. Sanchez L. Cole R. N. (2020). C9orf72-associated SMCR8 protein binds in the ubiquitin pathway and with proteins linked with neurological disease. *Acta Neuropathol. Commun.* 8:110. 10.1186/s40478-020-00982-x 32678027 PMC7364817

[B27] Grice G. L. Nathan J. A. (2016). The recognition of ubiquitinated proteins by the proteasome. *Cell Mol. Life Sci.* 73 3497–3506. 10.1007/s00018-016-2255-5 27137187 PMC4980412

[B28] Grumati P. Dikic I. Stolz A. (2018). ER-phagy at a glance. *J. Cell Sci.* 131:jcs217364. 10.1242/jcs.217364 30177506

[B29] Guerriero C. J. Brodsky J. L. (2012). The delicate balance between secreted protein folding and endoplasmic reticulum-associated degradation in human physiology. *Physiol. Rev.* 92 537–576. 10.1152/physrev.00027.2011 22535891 PMC4162396

[B30] Guo L. Giasson B. I. Glavis-Bloom A. Brewer M. D. Shorter J. Gitler A. D. (2014). A cellular system that degrades misfolded proteins and protects against neurodegeneration. *Mol. Cell* 55 15–30. 10.1016/j.molcel.2014.04.030 24882209 PMC4445634

[B31] Hartl F. U. Hayer-Hartl M. (2002). Molecular chaperones in the cytosol: from nascent chain to folded protein. *Science* 295 1852–1858. 10.1126/science.1068408 11884745

[B32] Hatakeyama S. (2011). TRIM proteins and cancer. *Nat. Rev. Cancer* 11 792–804. 10.1038/nrc3139 21979307

[B33] Hatakeyama S. (2017). TRIM family proteins: roles in Autophagy. Immunity, and Carcinogenesis. *Trends Biochem. Sci.* 42 297–311. 10.1016/j.tibs.2017.01.002 28118948

[B34] Hetz C. Chevet E. Oakes S. A. (2015). Proteostasis control by the unfolded protein response. *Nat. Cell Biol.* 17 829–838. 10.1038/ncb3184 26123108 PMC5546321

[B35] Hetz C. Saxena S. (2017). ER stress and the unfolded protein response in neurodegeneration. *Nat. Rev. Neurol.* 13 477–491. 10.1038/nrneurol.2017.99 28731040

[B36] Hiller M. M. Finger A. Schweiger M. Wolf D. H. (1996). ER degradation of a misfolded luminal protein by the cytosolic ubiquitin-proteasome pathway. *Science* 273 1725–1728. 8781238 10.1126/science.273.5282.1725

[B37] Hjerpe R. Rodriguez M. S. (2008). Alternative UPS drug targets upstream the 26S proteasome. *Int. J. Biochem. Cell Biol.* 40 1126–1140. 10.1016/j.biocel.2007.11.021 18203645

[B38] Horwich A. L. Weissman J. S. (1997). Deadly conformations–protein misfolding in prion disease. *Cell* 89 499–510. 10.1016/s0092-8674(00)80232-9 9160742

[B39] James L. C. Keeble A. H. Khan Z. Rhodes D. A. Trowsdale J. (2007). Structural basis for PRYSPRY-mediated tripartite motif (TRIM) protein function. *Proc. Natl. Acad. Sci. U.S.A.* 104 6200–6205. 10.1073/pnas.0609174104 17400754 PMC1851072

[B40] Janer A. Martin E. Muriel M. P. Latouche M. Fujigasaki H. Ruberg M. (2006). PML clastosomes prevent nuclear accumulation of mutant ataxin-7 and other polyglutamine proteins. *J. Cell Biol.* 174 65–76. 10.1083/jcb.200511045 16818720 PMC2064165

[B41] Jena K. K. Kolapalli S. P. Mehto S. Nath P. Das B. Sahoo P. K. (2018a). TRIM16 controls assembly and degradation of protein aggregates by modulating the p62-NRF2 axis and autophagy. *EMBO J.* 37:e98358. 10.15252/embj.201798358 30143514 PMC6138442

[B42] Jena K. K. Mehto S. Kolapalli S. P. Nath P. Chauhan S. Chauhan S. (2018b). TRIM16 employs NRF2, ubiquitin system and aggrephagy for safe disposal of stress-induced misfolded proteins. *Cell Stress* 2 365–367. 10.15698/cst2018.12.169 31225461 PMC6551674

[B43] Jena K. K. Mehto S. Kolapalli S. P. Nath P. Sahu R. Chauhan N. R. (2019). TRIM16 governs the biogenesis and disposal of stress-induced protein aggregates to evade cytotoxicity: implication for neurodegeneration and cancer. *Autophagy* 15 924–926. 10.1080/15548627.2019.1586251 30806139 PMC6526826

[B44] Ji C. H. Kim H. Y. Heo A. J. Lee S. H. Lee M. J. Kim S. B. (2019). The N-Degron Pathway Mediates ER-phagy. *Mol. Cell* 75 1058–1072.e1059. 10.1016/j.molcel.2019.06.028 31375263

[B45] Kaganovich D. Kopito R. Frydman J. (2008). Misfolded proteins partition between two distinct quality control compartments. *Nature* 454 1088–1095. 10.1038/nature07195 18756251 PMC2746971

[B46] Keeble A. H. Khan Z. Forster A. James L. C. (2008). TRIM21 is an IgG receptor that is structurally, thermodynamically, and kinetically conserved. *Proc. Natl. Acad. Sci. U.S.A.* 105 6045–6050. 10.1073/pnas.0800159105 18420815 PMC2329685

[B47] Kim H. Bhattacharya A. Qi L. (2015). Endoplasmic reticulum quality control in cancer: friend or foe. *Semin. Cancer Biol.* 33 25–33. 10.1016/j.semcancer.2015.02.003 25794824 PMC4523434

[B48] Kim Y. E. Hipp M. S. Bracher A. Hayer-Hartl M. Hartl F. U. (2013). Molecular chaperone functions in protein folding and proteostasis. *Annu. Rev. Biochem.* 82 323–355. 10.1146/annurev-biochem-060208-092442 23746257

[B49] Kimura T. Jain A. Choi S. W. Mandell M. A. Schroder K. Johansen T. (2015). TRIM-mediated precision autophagy targets cytoplasmic regulators of innate immunity. *J. Cell Biol.* 210 973–989. 10.1083/jcb.201503023 26347139 PMC4576868

[B50] Kimura T. Jia J. Kumar S. Choi S. W. Gu Y. Mudd M. (2017). Dedicated SNAREs and specialized TRIM cargo receptors mediate secretory autophagy. *EMBO J.* 36 42–60. 10.15252/embj.201695081 27932448 PMC5210154

[B51] Kondo A. Shahpasand K. Mannix R. Qiu J. Moncaster J. Chen C. H. (2015). Antibody against early driver of neurodegeneration cis P-tau blocks brain injury and tauopathy. *Nature* 523 431–436. 10.1038/nature14658 26176913 PMC4718588

[B52] Korolchuk V. I. Mansilla A. Menzies F. M. Rubinsztein D. C. (2009). Autophagy inhibition compromises degradation of ubiquitin-proteasome pathway substrates. *Mol. Cell* 33 517–527. 10.1016/j.molcel.2009.01.021 19250912 PMC2669153

[B53] Kwon Y. T. Ciechanover A. (2017). The Ubiquitin code in the ubiquitin-proteasome system and autophagy. *Trends Biochem. Sci.* 42 873–886. 10.1016/j.tibs.2017.09.002 28947091

[B54] Lee J. M. Choi S. S. Lee Y. H. Khim K. W. Yoon S. Kim B.-G. (2018). The E3 ubiquitin ligase TRIM25 regulates adipocyte differentiation via proteasome-mediated degradation of PPARγ. *Exp. Mol. Med.* 50:135. 10.1038/s12276-018-0162-6 30323259 PMC6189217

[B55] Lerner M. Corcoran M. Cepeda D. Nielsen M. L. Zubarev R. Ponten F. (2007). The RBCC gene RFP2 (Leu5) encodes a novel transmembrane E3 ubiquitin ligase involved in ERAD. *Mol. Biol. Cell* 18 1670–1682. 10.1091/mbc.e06-03-0248 17314412 PMC1855009

[B56] Li Y. Wu H. Wu W. Zhuo W. Liu W. Zhang Y. (2014). Structural insights into the TRIM family of ubiquitin E3 ligases. *Cell Res.* 24 762–765. 10.1038/cr.2014.46 24722452 PMC4042170

[B57] Liu Y. Tao S. Liao L. Li Y. Li H. Li Z. (2020). TRIM25 promotes the cell survival and growth of hepatocellular carcinoma through targeting Keap1-Nrf2 pathway. *Nat. Commun.* 11:348. 10.1038/s41467-019-14190-2 31953436 PMC6969153

[B58] Mandell M. A. Jain A. Arko-Mensah J. Chauhan S. Kimura T. Dinkins C. (2014). TRIM proteins regulate autophagy and can target autophagic substrates by direct recognition. *Dev. Cell* 30 394–409. 10.1016/j.devcel.2014.06.013 25127057 PMC4146662

[B59] McEwan W. A. Falcon B. Vaysburd M. Clift D. Oblak A. L. Ghetti B. (2017). Cytosolic Fc receptor TRIM21 inhibits seeded tau aggregation. *Proc. Natl. Acad. Sci.U.S.A.* 114 574–579. 10.1073/pnas.1607215114 28049840 PMC5255578

[B60] McEwan W. A. Hauler F. Williams C. R. Bidgood S. R. Mallery D. L. Crowther R. A. (2012). Regulation of virus neutralization and the persistent fraction by TRIM21. *J. Virol.* 86 8482–8491. 10.1128/jvi.00728-12 22647693 PMC3421726

[B61] Meroni G. Diez-Roux G. (2005). TRIM/RBCC, a novel class of ‘single protein RING finger’. E3 ubiquitin ligases. *Bioessays* 27 1147–1157. 10.1002/bies.20304 16237670

[B62] Mishra R. Upadhyay A. Prajapati V. K. Mishra A. (2018). Proteasome-mediated proteostasis: novel medicinal and pharmacological strategies for diseases. *Med. Res. Rev.* 38 1916–1973. 10.1002/med.21502 29719055

[B63] New J. Thomas S. M. (2019). Autophagy-dependent secretion: mechanism, factors secreted, and disease implications. *Autophagy* 15 1682–1693. 10.1080/15548627.2019.1596479 30894055 PMC6735501

[B64] Niikura T. Hashimoto Y. Tajima H. Ishizaka M. Yamagishi Y. Kawasumi M. (2003). A tripartite motif protein TRIM11 binds and destabilizes Humanin, a neuroprotective peptide against Alzheimer’s disease-relevant insults. *Eur. J. Neurosci.* 17 1150–1158. 10.1046/j.1460-9568.2003.02553.x 12670303

[B65] Nisole S. Maroui M. A. Mascle X. H. Aubry M. Chelbi-Alix M. K. (2013). Differential roles of PML isoforms. *Front. Oncol.* 3:125. 10.3389/fonc.2013.00125 23734343 PMC3660695

[B66] Nisole S. Stoye J. P. Saib A. (2005). TRIM family proteins: retroviral restriction and antiviral defence. *Nat. Rev. Microbiol.* 3 799–808. 10.1038/nrmicro1248 16175175

[B67] Ohtake F. Saeki Y. Ishido S. Kanno J. Tanaka K. (2016). The K48-K63 branched ubiquitin chain regulates NF-kappaB signaling. *Mol. Cell* 64 251–266. 10.1016/j.molcel.2016.09.014 27746020

[B68] Ozato K. Shin D. M. Chang T. H. Morse H. C. (2008). TRIM family proteins and their emerging roles in innate immunity. *Nat. Rev. Immunol.* 8 849–860. 10.1038/nri2413 18836477 PMC3433745

[B69] Pertel T. Hausmann S. Morger D. Zuger S. Guerra J. Lascano J. (2011). TRIM5 is an innate immune sensor for the retrovirus capsid lattice. *Nature* 472 361–365. 10.1038/nature09976 21512573 PMC3081621

[B70] Pineda C. T. Ramanathan S. Fon Tacer K. Weon J. L. Potts M. B. Ou Y. H. (2015). Degradation of AMPK by a cancer-specific ubiquitin ligase. *Cell* 160 715–728. 10.1016/j.cell.2015.01.034 25679763 PMC5629913

[B71] Powers E. T. Balch W. E. (2008). Costly mistakes: translational infidelity and protein homeostasis. *Cell* 134 204–206. 10.1016/j.cell.2008.07.005 18662533

[B72] Richter K. Haslbeck M. Buchner J. (2010). The heat shock response: life on the verge of death. *Mol. Cell* 40 253–266. 10.1016/j.molcel.2010.10.006 20965420

[B73] Ron D. Walter P. (2007). Signal integration in the endoplasmic reticulum unfolded protein response. *Nat. Rev. Mol. Cell Biol.* 8 519–529. 10.1038/nrm2199 17565364

[B74] Sadowski M. Suryadinata R. Tan A. R. Roesley S. N. Sarcevic B. (2012). Protein monoubiquitination and polyubiquitination generate structural diversity to control distinct biological processes. *IUBMB Life* 64 136–142. 10.1002/iub.589 22131221

[B75] Saibil H. (2013). Chaperone machines for protein folding, unfolding and disaggregation. *Nat. Rev. Mol. Cell Biol.* 14 630–642. 10.1038/nrm3658 24026055 PMC4340576

[B76] Sharma S. Chakraborty K. Müller B. K. Astola N. Tang Y.-C. Lamb D. C. (2008). Monitoring protein conformation along the pathway of chaperonin-assisted folding. *Cell* 133 142–153. 10.1016/j.cell.2008.01.048 18394994

[B77] Sontag E. M. Samant R. S. Frydman J. (2017). Mechanisms and functions of spatial protein quality control. *Annu. Rev. Biochem.* 86 97–122. 10.1146/annurev-biochem-060815-014616 28489421

[B78] Soto C. Pritzkow S. (2018). Protein misfolding, aggregation, and conformational strains in neurodegenerative diseases. *Nat. Neurosci.* 21 1332–1340. 10.1038/s41593-018-0235-9 30250260 PMC6432913

[B79] Stremlau M. Owens C. M. Perron M. J. Kiessling M. Autissier P. Sodroski J. (2004). The cytoplasmic body component TRIM5alpha restricts HIV-1 infection in Old World monkeys. *Nature* 427 848–853. 10.1038/nature02343 14985764

[B80] Sun D. Wu R. Zheng J. Li P. Yu L. (2018). Polyubiquitin chain-induced p62 phase separation drives autophagic cargo segregation. *Cell Res.* 28 405–415. 10.1038/s41422-018-0017-7 29507397 PMC5939046

[B81] Takahata M. Bohgaki M. Tsukiyama T. Kondo T. Asaka M. Hatakeyama S. (2008). Ro52 functionally interacts with IgG1 and regulates its quality control via the ERAD system. *Mol. Immunol.* 45 2045–2054. 10.1016/j.molimm.2007.10.023 18022694

[B82] Tomar D. Prajapati P. Sripada L. Singh K. Singh R. Singh A. K. (2013). TRIM13 regulates caspase-8 ubiquitination, translocation to autophagosomes and activation during ER stress induced cell death. *Biochim. Biophys. Acta* 1833 3134–3144. 10.1016/j.bbamcr.2013.08.021 24021263

[B83] Tomar D. Singh R. Singh A. K. Pandya C. D. Singh R. (2012). TRIM13 regulates ER stress induced autophagy and clonogenic ability of the cells. *Biochim. Biophys. Acta* 1823 316–326. 10.1016/j.bbamcr.2011.11.015 22178386

[B84] Tuoc T. C. Stoykova A. (2008). Trim11 modulates the function of neurogenic transcription factor Pax6 through ubiquitin-proteosome system. *Genes Dev.* 22 1972–1986. 10.1101/gad.471708 18628401 PMC2492742

[B85] Varshavsky A. (2017). The Ubiquitin system, autophagy, and regulated protein degradation. *Annu. Rev. Biochem.* 86 123–128. 10.1146/annurev-biochem-061516-044859 28654326

[B86] Versteeg G. A. Rajsbaum R. Sanchez-Aparicio M. T. Maestre A. M. Valdiviezo J. Shi M. (2013). The E3-ligase TRIM family of proteins regulates signaling pathways triggered by innate immune pattern-recognition receptors. *Immunity* 38 384–398. 10.1016/j.immuni.2012.11.013 23438823 PMC3584420

[B87] Wang M. Kaufman R. J. (2016). Protein misfolding in the endoplasmic reticulum as a conduit to human disease. *Nature* 529 326–335. 10.1038/nature17041 26791723

[B88] Wang P. Benhenda S. Wu H. Lallemand-Breitenbach V. Zhen T. Jollivet F. (2018). RING tetramerization is required for nuclear body biogenesis and PML sumoylation. *Nat. Commun.* 9:1277. 10.1038/s41467-018-03498-0 29599493 PMC5876331

[B89] Wang X. Shi W. Shi H. Lu S. Wang K. Sun C. (2016). TRIM11 overexpression promotes proliferation, migration and invasion of lung cancer cells. *J. Exp. Clin. Cancer Res.* 35:100. 10.1186/s13046-016-0379-y 27329103 PMC4915141

[B90] Wani W. Y. Boyer-Guittaut M. Dodson M. Chatham J. Darley-Usmar V. Zhang J. (2015). Regulation of autophagy by protein post-translational modification. *Lab Invest.* 95 14–25. 10.1038/labinvest.2014.131 25365205 PMC4454381

[B91] Wiseman R. L. Powers E. T. Buxbaum J. N. Kelly J. W. Balch W. E. (2007). An adaptable standard for protein export from the endoplasmic reticulum. *Cell* 131 809–821. 10.1016/j.cell.2007.10.025 18022373

[B92] Wong E. Cuervo A. M. (2010). Integration of clearance mechanisms: the proteasome and autophagy. *Cold Spring Harb. Perspect. Biol.* 2:a006734. 10.1101/cshperspect.a006734 21068151 PMC2982176

[B93] Wu J. Kaufman R. J. (2006). From acute ER stress to physiological roles of the unfolded protein response. *Cell Death Differ.* 13, 374–384. 10.1038/sj.cdd.4401840 16397578

[B94] Xu P. Duong D. M. Seyfried N. T. Cheng D. Xie Y. Robert J. (2009). Quantitative proteomics reveals the function of unconventional ubiquitin chains in proteasomal degradation. *Cell* 137 133–145. 10.1016/j.cell.2009.01.041 19345192 PMC2668214

[B95] Zheng N. Shabek N. (2017). Ubiquitin ligases: structure, function, and regulation. *Annu. Rev. Biochem.* 86 129–157. 10.1146/annurev-biochem-060815-014922 28375744

[B96] Zou W. Zhang D. E. (2006). The interferon-inducible ubiquitin-protein isopeptide ligase (E3) EFP also functions as an ISG15 E3 ligase. *J. Biol. Chem.* 281 3989–3994. 10.1074/jbc.M510787200 16352599

